# 2111: Insulin resistance patterns over 25-years of adulthood and nonalcoholic fatty liver disease in middle age: The Coronary Artery Risk Development in Young Adults (CARDIA) Study

**DOI:** 10.1017/cts.2017.94

**Published:** 2018-05-10

**Authors:** Lisa B. VanWagner, Michael Bancks, Hongyan Ning, Juned Siddique, Cora Lewis, John Jeffrey Carr, Miriam Vos, Elizabeth Speliotes, Norah Terrault, Mary E. Rinella, Norrina B. Allen, Donald Lloyd-Jones

**Affiliations:** Northwestern University, Evanston, IL, USA

## Abstract

OBJECTIVES/SPECIFIC AIMS: Nonalcoholic fatty liver disease (NAFLD) is the most common cause of liver disease in the United States and increases risk for cirrhosis and liver cancer. Identifying modifiable risk factors for NAFLD could allow better targeting of prevention programs. Insulin resistance (IR) plays a significant role in the development and progression of NAFLD. IR is also an important precursor to the development of type 2 diabetes (T2DM). However, the development and duration of IR during young adulthood and its association with NAFLD and T2DM in midlife is unclear. To test whether trajectories of IR using homeostatic model assessment (HOMA-IR) change throughout early adulthood are associated with risk of prevalent NAFLD and T2DM among persons with NAFLD in midlife independent of current or baseline HOMA-IR. METHODS/STUDY POPULATION: Participants from the CARDIA study, a prospective multicenter population-based biracial cohort of adults (baseline age 18–30 years), underwent HOMA-IR measurement (≥8 h fasting and not pregnant) at baseline (1985–1986) and follow-up exam years 7, 10, 15, 20, and 25. At Year 25 (Y25, 2010–2011), liver fat was assessed by noncontrast computed tomography (CT). NAFLD was defined as CT liver attenuation <51 Hounsfield Units after exclusion of other causes of liver fat (alcohol/hepatitis/medications). Latent mixture modeling was used to identify 25-year trajectories in HOMA-IR over time. Multivariable logistic regression models were used to assess associations between HOMA-IR trajectory groups and prevalent NAFLD with adjustment for baseline or Y25 HOMA-IR. RESULTS/ANTICIPATED RESULTS: Among 3060 participants, we identified 3 distinct trajectory groups for HOMA-IR for individuals free from diabetes in middle adulthood: qualitatively low-stable (46.7% of the cohort), moderate-increasing (42.0%), and high-increasing (11.3%) with a NAFLD prevalence at Y25 of: 8.3%, 33.4%, and 63.5%, respectively (*p*-trend<0.0001). After adjustment for confounders (baseline smoking status, alcohol use, body mass index, physical activity score, systolic blood pressure, antihypertensive medication use, and total/HDL cholesterol ratio) and baseline HOMA-IR, increasing HOMA-IR trajectories were associated with greater NAFLD prevalence compared with the low-stable trajectory group [odds ratio (95% CI): 5.8 (4.3–7.9) and 22.3 (14.2–34.9) for moderate and high, respectively]. These associations were attenuated, but remained significant, even after controlling for current Y25 HOMA-IR [OR=3.6 (2.6–5.0) for moderate and 5.9 (3.4–10.3) for high (referent: low)]. Among participants with NAFLD (n=511), high-increasing HOMA-IR trajectory was associated with greater prevalent [OR=6.5 (1.6–25.7)] and incident [OR=8.7 (2.2–34.4)] T2DM at Y25 independent of confounders and Y25 HOMA-IR (referent: low-stable). DISCUSSION/SIGNIFICANCE OF IMPACT: In this community-based sample of individuals free from diabetes at baseline, an increasing HOMA-IR trajectory through young adulthood was associated with greater NAFLD prevalence in midlife. Knowledge of changes in IR throughout adulthood provides new information on the risk of T2DM among persons with NAFLD in midlife independent of current level of IR. These findings highlight early identification of increasing IR as a potential target for primary prevention of T2DM in the setting of NAFLD.

